# Subthalamic nucleus deep brain stimulation induces nigrostriatal dopaminergic plasticity in a stable rat model of Parkinson’s disease

**DOI:** 10.1097/WNR.0000000000001917

**Published:** 2023-05-20

**Authors:** Charlotte Helf, Maria Kober, Franz Markert, Jennifer Lanto, Leonie Overhoff, Kathrin Badstübner-Meeske, Alexander Storch, Mareike Fauser

**Affiliations:** a Department of Neurology, University of Rostock; b German Centre for Neurodegenerative Diseases (DZNE) Rostock/Greifswald, Rostock, Germany

**Keywords:** c-Fos, dopaminergic neurons, high-frequency stimulation, 6-hydroxydopamine, immediate early genes, mesolimbic system, neurorestoration, nigrostriatal system, Parkinson’s disease, ventral tegmental area

## Abstract

Deep brain stimulation (DBS) of the subthalamic nucleus (STN) has been a highly effective treatment option for middle to late stage Parkinson’s disease for decades. Though, the underlying mechanisms of action, particularly effects on the cellular level, remain in part unclear. In the context of identifying disease-modifying effects of STN-DBS by prompting cellular plasticity in midbrain dopaminergic systems, we analyzed neuronal tyrosine hydroxylase and c-Fos expression in the substantia nigra pars compacta (SNpc) and ventral tegmental area (VTA).

**Methods:**

We applied 1 week of continuous unilateral STN-DBS in a group of stable 6-hydroxydopamine (6-OHDA) hemiparkinsonian rats (STN_STIM_) in comparison to a 6-OHDA control group (STN_SHAM_). Immunohistochemistry identified NeuN^+^, tyrosine hydroxylase^+^ and c-Fos^+^ cells within the SNpc and VTA.

**Results:**

After 1 week, rats in the STN_STIM_ group had 3.5-fold more tyrosine hydroxylase^+^ neurons within the SNpc (*P* = 0.010) but not in the VTA compared to sham controls. There was no difference in basal cell activity as indicated by c-Fos expression in both midbrain dopaminergic systems.

**Conclusion:**

Our data support a neurorestorative effect of STN-DBS in the nigrostriatal dopaminergic system already after 7 days of continuous STN-DBS in the stable Parkinson’s disease rat model without affecting basal cell activity.

## Introduction

Deep brain stimulation (DBS) of the subthalamic nucleus (STN) is a highly effective treatment option in middle to late stage Parkinson’s disease, offering long-lasting motor symptom benefits for Parkinson’s disease patients suffering from motor fluctuations and dyskinesia [1]. While motor symptoms improve within seconds to a few hours after DBS onset, new data suggest that long-term DBS in Parkinson’s disease patients also results in a noticeable, though delayed decline of various non-motor symptoms (NMSs), such as fatigue, hallucinations and urinary dysfunction [2].

The different dynamics of motor and non-motor improvements in Parkinson’s disease patients with DBS therapy might be associated with distinct underlying mechanisms of action of STN-DBS. The almost immediate improvement of motor symptoms within minutes to hours to days after STN-DBS onset most likely corresponds to a disruption of pathological network activity [3], such as suppression of pathological beta oscillations [4,5]. In contrast, the mechanisms behind the delayed reduction of NMSs have not been clarified so far. In this context, the impact of STN-DBS in Parkinson’s disease on the mesolimbic dopaminergic decline, which is considered a critical factor in neuropsychiatric manifestations of the disease, remains to be understood [6–8]. In a recent study, we determined the actions of long-term STN-DBS over 5–6 weeks on the midbrain dopaminergic deficit and detected neurorestorative effects of STN-DBS not only in nigrostriatal dopaminergic neurons but also in the ventral tegmental area (VTA) [9].

The present study investigates the effects of short-term STN-DBS (1 week) on enzymatic and cellular activity in midbrain dopaminergic systems in the 6-hydroxydopamine (6-OHDA) hemiparkinsonian rat model, in which we analyzed tyrosine hydroxylase and c-Fos expression in the substantia nigra pars compacta (SNpc) and VTA. Tyrosine hydroxylase is the rate-limiting enzyme of dopamine synthesis and shows dysregulation and degradation in Parkinson’s disease [10,11]. In addition, tyrosine hydroxylase concentration decreases markedly after 6-OHDA injection, resulting in the model’s typical manifestation of Parkinson’s disease motor symptoms [12]. C-Fos is a marker for neuronal and synaptic activity and has previously been used in rodent 6-OHDA models to assess the effects of short-term DBS from minutes up to four hours [13–15]. In the present study, we characterized STN-DBS effects on the nigrostriatal and mesolimbic dopaminergic systems after 1 week of continuous DBS to describe the dynamics of neurorestorative effects in the 6-OHDA Parkinson’s disease rat model. In addition, we investigated c-Fos expression as an indicator of long-term DBS-induced neuronal activity alterations and histological marker of effective stimulation.

## Methods

### Animals

All procedures were permitted by responsible authorities (Landesamt für Landwirtschaft, Lebensmittelsicherheit und Fischerei, Mecklenburg-Vorpommern, Germany) and carried out in line with Animal Research: Reporting of In Vivo Experiments guidelines and the European Union (EU) Directive 2010/63/EU. We used male rats (265–360 g; purchased from Charles River Laboratories, Sulzfeld, Germany). All rats (*n* = 8) underwent right-sided unilateral 6-OHDA lesioning to generate a reliable dopaminergic degeneration in the ventral midbrain, including the VTA, as described previously in detail [9,16]. Briefly, rats were anesthetized with isoflurane followed by weight-adapted ketamine/xylazine administration and injected with a total of 24 µg of 6-OHDA dissolved in 4 µl 0.1 M citrate buffer into the right median forebrain bundle according to the rat brain atlas [17] using a stereotactic frame (Stoelting Neuroscience, Dublin, Ireland) at the following coordinates (relative to bregma and dura): anterior–posterior −2.3, medial-lateral +1.5 and dorsal-ventral −8.5. Successful lesioning was quantified by apomorphine-induced rotational behavior 6 weeks after 6-OHDA treatment; then, all animals were divided into two closely matched groups [STN_STIM_ (mean ± SEM): 11.2 ± 3.1 rotations/min; STN_SHAM_: 13.3 ± 3.8 rotations/min; *P* = 0.671 from unpaired two-sided *t*-test, *n* = 4]. Short-term (4 h) DBS-treated animals were gathered from previous unpublished cohorts and received identical treatments except for DBS stimulation paradigms.

### Surgery

**Fig. 1:**
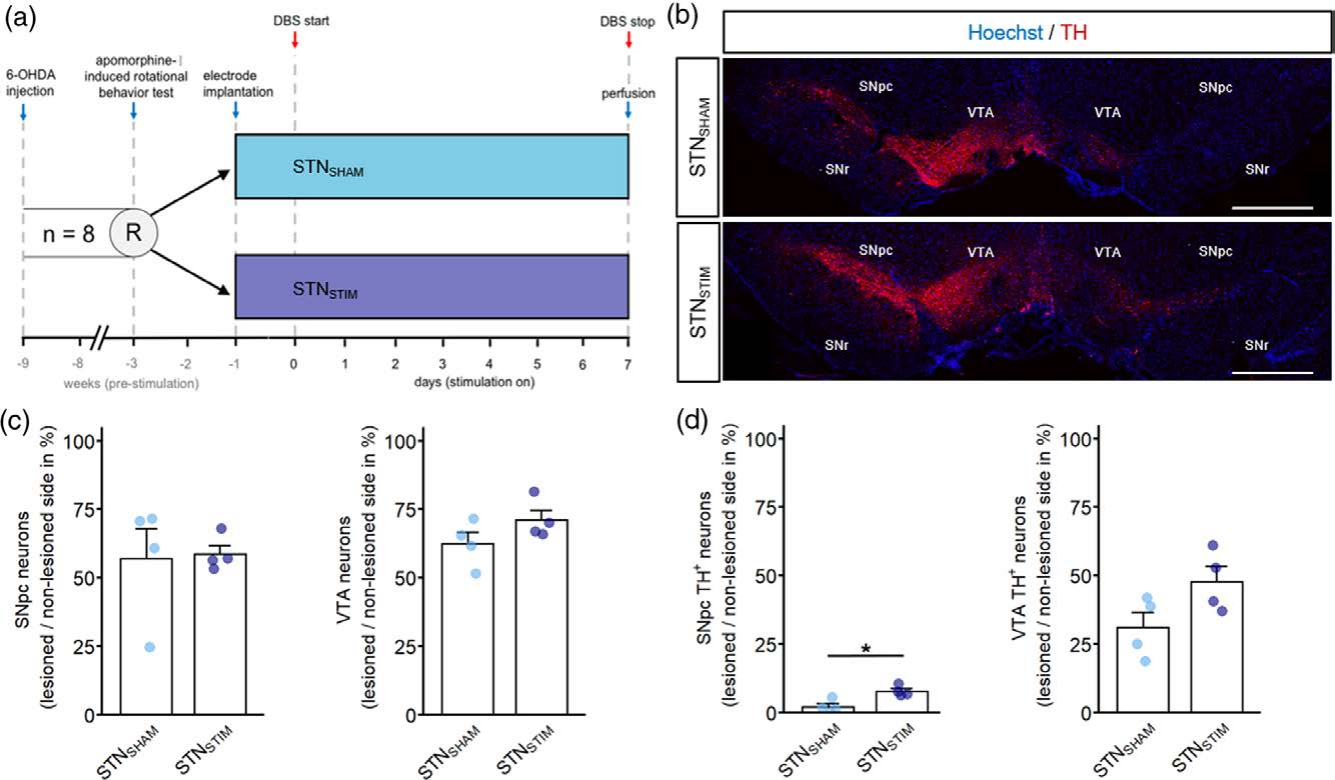
(a) Experimental design. (b) Representative images of the midbrain dopaminergic systems after right-sided 6-OHDA lesioning with consecutive dopaminergic degeneration; cell nuclei are Hoechst^+^ (blue) and dopaminergic cells are tyrosine hydroxylase^+^ (red). Scale bar 1000 µm. (c) Quantitative analysis of NeuN^+^ neurons in the SNpc (left panel) and VTA (right panel). (d) Quantitative analysis of tyrosine hydroxylase^+^ dopaminergic neurons in the SNpc (left panel) and VTA (right panel). Data are presented as mean ± SEM. for the lesioned hemispheres normalized to non-lesioned hemispheres. *P < 0.05 were deemed significant after two-sided *t*-test. Abbreviations: 6-OHDA, 6-hydroxydopamine; DBS, deep brain stimulation; SNpc, substantia nigra pars compacta; SNr, substantia nigra pars reticularis; STN, subthalamic nucleus; VTA, ventral tegmental area.

We implanted commercial unipolar platinum–iridium electrodes (Microprobes for Life Sciences, Maryland, USA) into the right STN of all animals 8 weeks after initial lesioning. Rats were left to recover for at least 7 days until stimulation onset (STN_STIM_; *n* = 4) or sham stimulation (STN_SHAM_; *n* = 4; see Fig. 1a for experimental setups). We used an external high-frequency stimulator carried in a rodent backpack with the following stimulation parameters: 100 µA, 130 Hz, 60 µs for 1 week [16]. For short-term stimulation, chronic DBS was switched off 28 h before the end of the study and again switched on 4 h before exit. Sham-stimulated animals received identical treatments, including dummy stimulators in their backpacks. After 1 week of stimulation, all animals were anesthetized and transcardially perfused with 4% paraformaldehyde. Brains were harvested and immersed in 4% paraformaldehyde for an additional 24 h, snap-frozen and stored at −80 °C.

### Immunohistochemistry

For immunohistochemistry, brains were cut into 30 µm thin coronal sections using a cryomicrotome (CM3050 S; Leica Biosystems, Nussloch, Germany). We performed triple immunostainings as described in [18] of every sixth section with the following primary antibodies: mouse anti-tyrosine hydroxylase (1 : 1000, RRID: AB_477560; Merck KGaA, Frankfurt on Main, Germany), chicken anti-NeuN (1 : 1000, RRID: AB_11205760; Sigma-Aldrich Corporate, St. Louis, USA) and rabbit anti-c-Fos (1 : 1400, RRID: AB_2247211, both from Sigma-Aldrich Corporate, St. Louis, USA) as well as specific secondary fluorescent antibodies: donkey anti-rabbit Alexa Fluor 488, donkey anti-chicken Cy3 and donkey anti-mouse Alexa Fluor 647 (all 1 : 500, all from Molecular Probes, Invitrogen, Germany). Cell nuclei were counterstained with Hoechst 33342 (Sigma Aldrich, Germany).

### Imaging, quantification and statistics

Respective SNpc and VTA sections were imaged and quantified using a motorized Axio.Observer.Z1 and ZEN Blue 2.3 software with Tiles and Position Module (Carl Zeiss, Oberkochen, Germany) according to rat brain atlas between bregma coordinates −5.04 and −5.76 [17]. For quantitative histology, we defined a region of interest (ROI) and counted all tyrosine hydroxylase^+^, c-Fos^high^ and c-Fos^low^ cells. c-Fos^high^ cells were defined as cells with an intense, perinuclear c-Fos staining as described previously [15,18], while c-Fos^low^ cells demonstrated a more scattered, punctiform c-Fos staining (see Supplementary Figure 2, Supplemental Digital Content, http://links.lww.com/WNR/A701 for details). For counting NeuN^+^ neurons and Hoechst^+^ total cells, we placed two (SNpc) or four (VTA) counting squares with a set volume of 100 × 100 × 30 µm^3^ randomly within the ROI. Tyrosine hydroxylase^+^ fibers were quantified in coronal sections corresponding to three antero-posterior levels of the striatum (+0.7 to +1.7 mm anterior to bregma [17]), more precisely in the dorsal striatum (caudate nucleus-putamen; CPu) as the main target region of SNpc neurons and the ventral striatum (core region of the *Nucleus accumbens*; NAc) as the target region of VTA dopaminergic neurons. The optical density (OD) value obtained for an unlabeled area (anterior commissure; ACo) was used as background and was subtracted from each of the OD values measured (as described in [9]). Shapiro–Wilk and Levene’s tests were applied to test for normal distribution and homogeneity of variances. We used unpaired *t*-test or Welch tests to determine mean differences between groups. All data are presented as mean ± SEM. Statistical significance was set at *P* < 0.05.

## Results

To determine the possible effects of 7 days of continuous STN-DBS (STN_STIM_) or respective sham stimulation (STN_SHAM_) on total neuron counts and dopaminergic cell numbers in midbrain dopaminergic systems, we used quantitative analyses of SNpc and VTA neurons in STN_SHAM_ and STN_STIM_ hemiparkinsonian rats (Figs. 1a and b). All data are presented as relative numbers compared to the contralateral non-lesioned, non-stimulated hemisphere. We found no significant alterations in the numbers of NeuN^+^ neurons neither in the SNpc nor in the VTA in STN_SHAM_ compared to STN_STIM_ animals (Fig. 1c). Relative amounts of NeuN^+^ neurons (compared to non-lesioned hemispheres) were 56.9 ± 11.0% in STN_SHAM_ and 58.7 ± 3.2% in STN_STIM_ animals in the SNpc (*P* = 0.883), and 62.5%±4.2% in STN_SHAM_ and 71.1 ± 3.6% in STN_STIM_ animals in the VTA (*P* = 0.169). However, relative amounts of tyrosine hydroxylase^+^ dopaminergic neurons were increased by 3.5-fold in the SNpc in STN_STIM_ animals with 7.8 ± 1.0% compared to the STN_SHAM_ cohort with 2.1 ± 1.2% (*P* = 0.010). In contrast, there was no significant alteration in the VTA, where we found 31.1 ± 5.5% of tyrosine hydroxylase^+^ neurons in STN_SHAM_ compared to 47.8 ± 5.5% in STN_STIM_ animals (*P* = 0.075; Fig. 1d).

Regarding dopaminergic innervation of the respective target regions, we quantified ODs of dopaminergic fibers within the CPu and NAc. Within the CPu, relative tyrosine hydroxylase ODs (compared to non-lesioned hemispheres) were 2.0 ± 1.0% in STN_SHAM_ and 3.3 ± 2.1% in STN_STIM_ animals (*P* = 0.56) relative to contralateral, non-lesioned hemispheres. In the NAc, we found 4.4 ± 1.2% in STN_SHAM_ and 7.4 ± 0.5% in STN_STIM_ animals (*P* = 0.11; Supplementary Figure S1, Supplemental Digital Content, http://links.lww.com/WNR/A701).

**Fig. 2:**
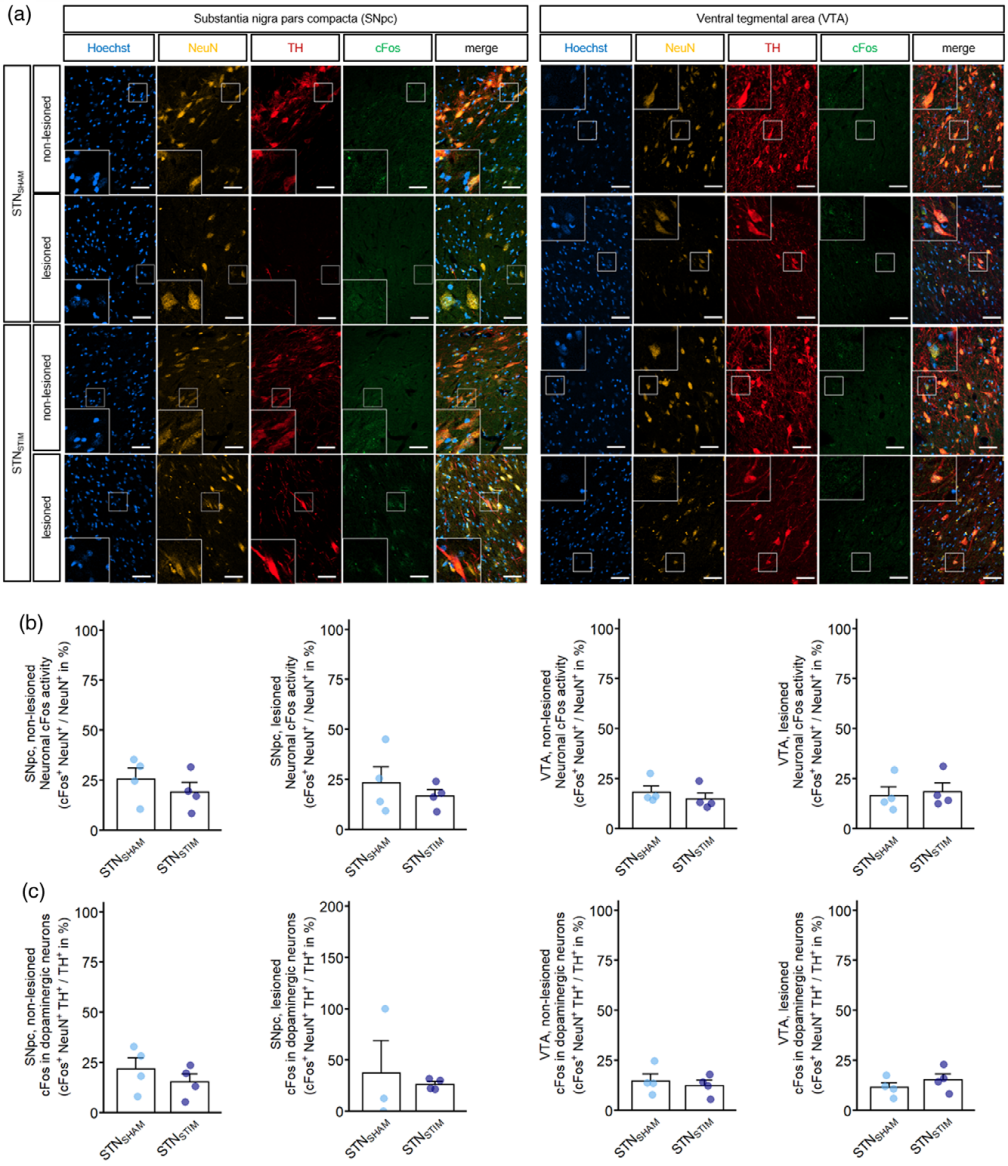
(a) Representative images of both lesioned and non-lesioned hemispheres of substantia nigra pars compacta (SNpc) and ventral tegmental area (VTA) of STN_STIM_ and STN_SHAM_ animals with Hoechst^+^ (blue) total cell counts (first column), NeuN^+^ (orange) neurons (second column), tyrosine hydroxylase^+^ (red) dopaminergic neurons (third column) and c-Fos^+^ (green) activated cells (fourth column). The lower left corner represents an enlarged image section from the indicated square. Scale bar represents 50 µm. (b) Comparative analyses of basal active c-Fos^+^ neurons in the non-lesioned (first column) and lesioned (second column) SNpc as well as in the non-lesioned (third column) and lesioned (fourth column) VTA in STN_STIM_ and STN_SHAM_ animals. Data are normalized to total NeuN^+^ neuron counts. (c) Comparative analyses of basal active c-Fos^+^ tyrosine hydroxylase^+^ dopaminergic neurons in the non-lesioned (first column) and lesioned (second column) SNpc as well as in the non-lesioned (third column) and lesioned (fourth column) VTA in STN_STIM_ and STN_SHAM_ animals. Data are normalized to total tyrosine hydroxylase^+^ neuron counts. Data are presented as mean ± SEM. *P* < 0.05 were deemed significant after two-sided t-test or Welch test. Abbreviations: NeuN, neuronal nuclei antigen; STN, subthalamic nucleus.

Furthermore, we used c-Fos immunohistochemistry as an indicator of neuronal activation. We could not detect c-Fos^high^ cells with intense, perinuclear c-Fos staining [15,18] in our cohort after 7 days of STN-DBS, but we were able to show c-Fos^high^ cells in our additional short-term 4 h STN-DBS stimulated animals. However, we could determine cells with specific but low intense c-Fos staining considered to represent basal c-Fos activity (c-Fos^low^; Supplementary Figure S2, Supplemental Digital Content, http://links.lww.com/WNR/A701). There was no significant effect of STN-DBS on the amount of c-Fos^low^ neurons in both hemispheres (Fig. 2b; for quantitative data and statistics, refer to Supplementary Table S1, Supplemental Digital Content, http://links.lww.com/WNR/A701). In addition, we analyzed the amounts of c-Fos^low^/tyrosine hydroxylase^+^ dopaminergic neurons and did not find any STN-DBS effects on c-Fos^low^/tyrosine hydroxylase^+^ dopaminergic neuron counts on both sides in both SNpc and VTA (Fig. 2c; for quantitative data and statistics, refer to Supplementary Table S1, Supplemental Digital Content, http://links.lww.com/WNR/A701).

## Discussion

In the present study, continuous unilateral STN-DBS for 7 days induced cellular plasticity in nigrostriatal dopaminergic neurons since it led to a gain of tyrosine hydroxylase^+^ dopaminergic neurons in the SNpc in the 6-OHDA parkinsonian rat model with stable dopaminergic neurodegeneration. In contrast, it did neither induce alterations in adjacent VTA dopaminergic neurons nor in dopaminergic fiber densities in the striatum. The significant increase of tyrosine hydroxylase^+^ nigrostriatal neurons without concordant alterations in total neuron counts supports previous findings of preserved tyrosine hydroxylase expression after unilateral STN-DBS and formerly reported neuroprotective effects of unilateral STN-DBS in the case of acute dopaminergic toxicity [19–21]. The lack of a concordant increase in striatal dopaminergic innervation might be attributed to the short STN-DBS duration of only 1 week. While most studies utilized DBS during the ongoing dopaminergic degeneration after 6-OHDA lesioning, we here applied DBS at least 9 weeks after initial lesioning to represent a stable dopaminergic deficit more closely resembling the human disease at the time point of DBS initiation [9].

In contrast to the present results, prolonged *unilateral* STN-DBS in a previous study with similar DBS conditions over 3–6 weeks did not alter SNpc dopaminergic neuron counts, but instead increased the number of dopaminergic neurons in the. On the other hand, long-term *bilateral* STN-DBS raised both VTA and SNpc dopaminergic neuron counts without affecting total neuron counts in these regions and also led to an increased dopaminergic innervation of the dorsal and ventral striatum [9]. We already excluded local neurogenesis as the source of increased numbers of tyrosine hydroxylase^+^ neurons, though a rescue of the dopaminergic phenotype in toxically damaged neurons seems a plausible mechanism as already described for both STN lesioning and STN-DBS [21–23].

The mechanisms mediating these differential effects of STN-DBS are yet unclear. In addition to the factors already mentioned (duration of stimulation and unilateral vs. bilateral stimulation), the extent of the dopaminergic lesion varied significantly between the above-mentioned studies [19,24]. While the present studies used rats with severe dopaminergic lesions (2 ± 1% of contralateral SNpc dopaminergic neuron counts in STN_SHAM_ animals), our previous study was conducted in moderately lesioned animals (33 ± 8% of dopaminergic neurons were preserved on the lesioned as compared to non-lesioned side, *P* = 0.029, Mann–Whitney *U*-test; *n* = 4 per group [9]). Whether the extent of the dopaminergic lesion actually interferes with STN-DBS mediated dopaminergic plasticity remains enigmatic. Additional mechanisms that are involved in the degenerative process, such as inflammation [25], are unlikely responsible due to the stable nature of the dopaminergic degeneration in our model [9].

In contrast to other studies on DBS-induced neuronal activation [18], we could not determine an evenly distributed c-Fos expression along the entire nuclear surface, but more a scattered c-Fos attachment possibly related to basal cell activity. Most studies evaluate c-Fos expression after hours and not days of STN-DBS since the c-Fos peak typically arises shortly after stimulation [13–15]. However, persistent alterations in basal c-Fos activity due to downregulation occur up to 3 weeks after the initial stimulus [26,27]. Still, continuous STN-DBS does not seem to persistently alter such basal cell activity, neither in SNpc nor in VTA, though strong direct (glutamatergic) projections from the STN to the ipsilateral SNpc and VTA are described in the literature [28–30]. Therefore, these data suggest that persistent STN-DBS-mediated activity of the dopaminergic neurons—at least that leading to c-Fos expression, is not involved in STN-DBS-induced dopaminergic plasticity in parkinsonian rats.

Our study is limited regarding its small animal numbers per group due to the very high experimental effort in such cohorts. Yet, the final group sizes are in the range of most similar experimental DBS studies in small animal models [19,24]. We tried to minimize this limitation by using closely matched groups according to their apomorphine-induced rotational behavior, which exhibits modest correlations between SNpc dopaminergic cell loss and test results [31]. Nevertheless, quantitative behavioral assessments for mesolimbic dopaminergic dysfunction independent of motor function are currently unavailable.

Together, we interpret our results as neurorestorative effects by a rescue of the dopaminergic phenotype of remaining midbrain neurons in parkinsonian rats. Although we cannot conclude on the cellular mechanisms underlying the restorative action of STN-DBS, our study shows that STN-DBS has positive effects on midbrain dopaminergic neurons. Nevertheless, further research is necessary to provide more information about the relationship between STN-DBS and its restorative activity with respect to its time course, severity of dopaminergic lesion and putative importance of bilateral stimulation for sustained neurorestorative effects.

## Acknowledgements

The authors thank Uta Naumann for her help with immunohistochemistry and Vivien Charlotte Retzlaff for her help with DBS electrode localization. C.H. created the figures and also included them in her M.D. thesis. This work was supported by the Deutsche Forschungsgemeinschaft (DFG) through the Collaborative Research Centre CRC 1270 ‘Electrically Active Implants’ (DFG; SFB 1270/1,2 – 299150580) to A.S. M.F. was supported by the CRC 1270 ‘Electrically Active Implants’ (DFG; SFB 1270/1 – 299150580) through the rotation position program for clinician scientists and by the Else Hirschberg Women´s Advancement Program of the University Medical Centre Rostock. C.H. and L.O. were supported by the CRC 1270 ‘Electrically Active Implants’ (DFG; SFB 1270/1,2 – 299150580) through an Integrated Research Training Program fellowship.

### Conflicts of interest

There are no conflicts of interest.

## Supplementary Material


