# Supplementary material for: Subthalamic nucleus deep brain stimulation induces nigrostriatal dopaminergic plasticity in a stable rat model of Parkinson’s disease
Source: Neuroreport. 2023 May 20;34(10):506–11. doi: 10.1097/WNR.0000000000001917 (PMC10234325; doi:10.1097/WNR.0000000000001917)
Supplement: Supplementary file 1 [file nr-34-506-s001.pdf]

for submission to Neuroreport

Subthalamic nucleus deep brain stimulation induces nigrostriatal dopaminergic plasticity in a stable rat model of Parkinson's disease

Charlotte Helf,[a] Maria Kober,[a] Franz Markert,[a] Jennifer Lanto,[a] Leonie Overhoff, [a] Kathrin Badstübner-Meeske,[a] Alexander Storch[a,b] and Mareike Fauser [a]

Supplemental Materials

- **Figure S1.** Influence of one week of STN-DBS on striatal dopaminergic neurotransmission.
- **Figure S2.** Intensity of c-Fos immunostainings depending on STN-DBS durations.
- **Table S1.** Quantitative data and statistics of c-Fos immunohistochemistry.

Supplementary figures:

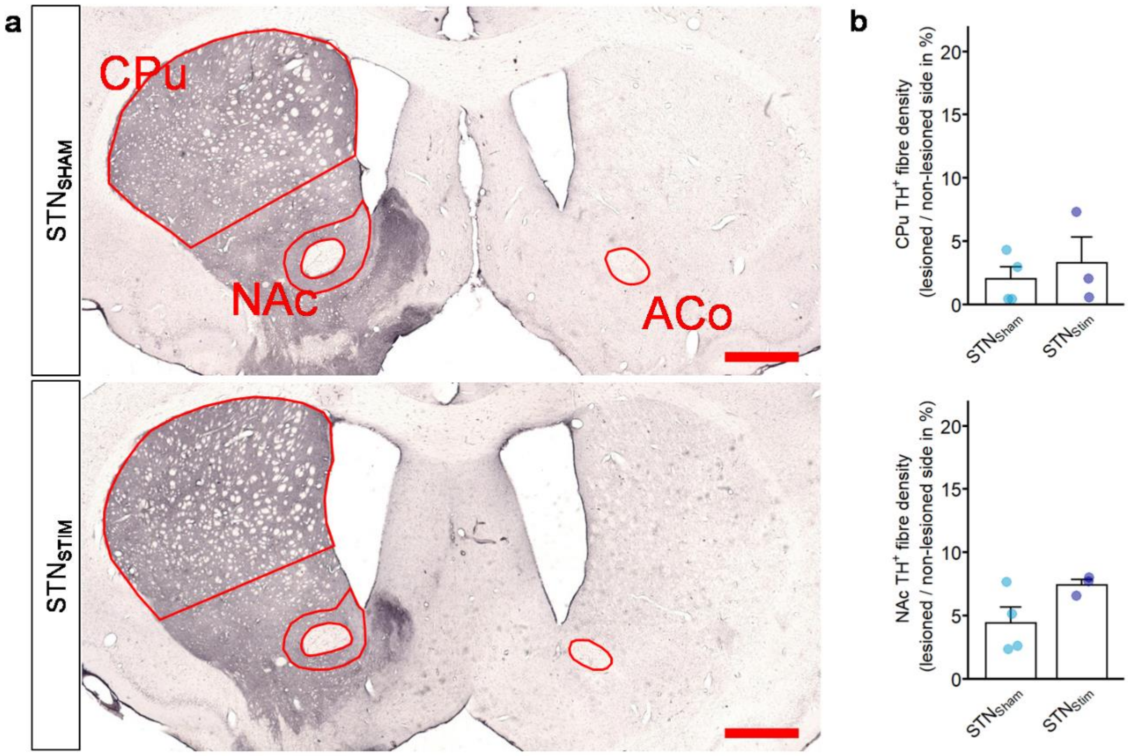

Supplementary Figure S1: (a) Representative immunohistological analyses of tyrosine hydroxylase (TH) stainings in the striatum of hemiparkinsonian rats after seven days of unilateral, right-sided STN-DBS. Marked regions are the dorsal striatum (= Caudate nucleus putamen; CPu) and the *Nucleus accumbens core region* (NAc) as the main target areas of the midbrain dopaminergic systems and the anterior commissure (ACo) as the reference region (white matter). Scale bars, 1000 µm. **(b)** Densiometric analysis of TH^+ fibre densities within the CPu as the main target region of *substantia nigra* neurons and the NAc as the main target region of neurons in the ventral tegmental area showed no effects of STN-DBS in lesioned hemispheres (normalized to respective non-lesioned hemispheres). **Abbreviations:** STN - subthalamic nucleus; TH – tyrosine hydroxylase.

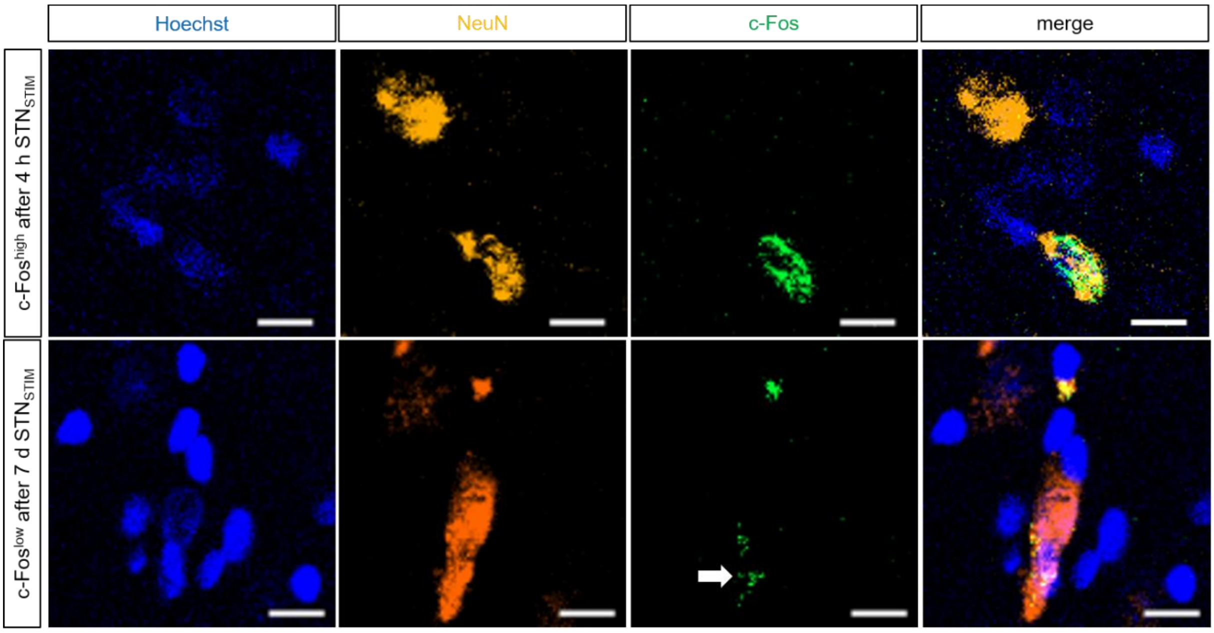

Supplementary Figure S2: Immunohistochemical c-Fos staining reveals persistent basal c-Fos expression after 7 days of continuous STN-DBS (white arrow; lower panel; c-Fos^{low}), which differs from more widespread expression after short-term stimulation after 4h (upper panel; c-Fos^{high}). Scale bar 10 µm. *Abbreviation*: STN - subthalamic nucleus.

	**Non-lesioned side**				**Lesioned side**		
	STN_{SHAM}	STN_{STIM}	*P*-value		STN_{SHAM}	STN_{STIM}	*P*-value
**c-Fos^{low} cells (% of cells)**							
**SNpc**	25.6±5.5%	19.1±4.8%	0.404		23.4±7.9%	16.8±3.1%	0.467
**VTA**	18.5±6.4%	15.3±5.9%	0.484		16.5±8.7%	18.5±8.6%	0.755
**c-Fos^{low}/TH^{+} neurons (% of all TH^{+} neurons)**							
**SNpc**	21.8±5.5%	15.4±4.0%	0.382		37.5±31.5%	26.4±2.7%	0.692
**VTA**	14.8±3.52%	12.5±2.6%	0.606		11.6±2.4%	15.4±3.0%	0.355

Supplementary Table S1. Quantitative data and statistics of c-Fos immunohistochemistry. Data are presented as mean values ± S.E.M. (n=3 or 4). *P*-values are from unpaired two-sided t-tests or Welch tests as appropriate to determine mean differences between groups. **Abbreviations:** STN - subthalamic nucleus; TH - tyrosine hydroxylase; SNpc - Substantia nigra pars compacta; VTA - ventral tegmental area.
